# New and Re-Emerging Infectious Diseases

**DOI:** 10.3201/eid1010.040531

**Published:** 2004-10

**Authors:** Roberto Docampo

**Affiliations:** * University of Illinois at Urbana-Champaign, Illinois, USA

**Keywords:** yersinia, mycoplasma, microsporidia, cryptococcus, trypanosoma, entamoeba, shigella, staphylococcus, SARS

The seventh annual Conference on New and Re-Emerging Infectious Diseases, April 15–16, 2004, was hosted by the Center for Zoonoses Research and the College of Veterinary Medicine at the University of Illinois at Urbana-Champaign. The conference featured eight speakers and 31 poster presentations.

The conference was opened with a presentation on *Yersinia enterocolitica*, a gram-negative enteric human pathogen that causes enterocolitis. Invasin is the primary requirement for efficient translocation of the bacteria across the intestinal epithelium; the identification of both positive and negative regulators of its expression have been identified.

The emergence of *Mycoplasma gallisepticum*, which causes severe conjunctivitis in house finches, was described. The strain of *M. gallisepticum* that affects house finches, a new host recently introduced to North America, is a novel strain that emerged recently.

Current therapies for microsporidiosis, a serious opportunistic infection in persons with AIDS, organ transplant recipients, children, travelers, contact lens wearers, and the elderly, were reviewed.

Studies on the human fungal pathogen *Cryptococcus neoformans*, which causes life-threatening infections of the central nervous system, most commonly in immunocompromised hosts, were reported. The studies focused on signaling cascades that govern virulence and an unusual mating type locus linked to differentiation and virulence.

It was shown that release of the variant surface antigens of African trypanosomes, agents of a reemerging infectious disease in sub-Saharan Africa, occurs not only by proteolysis but also by glycosylphosphatidylinositol-anchor hydrolysis through a phospholipase present in the parasite surface.

To characterize the human colonic response to *Shigella* and *Entamoeba histolytica* at the molecular level, differential transcription of nearly 40,000 human genes in sections of human colonic xenografts that had been infected with *Shigella flexneri* or *E. histolytica* was measured. The results indicated increased expression of genes encoding proteins involved in stress, hypoxic responses, immune and inflammatory responses, responses to tissue injury and tissue repair, cytokines, and chemokines.

Studies on sortases, membrane-anchored transpeptidases that cleave surface proteins, were described. Because sortases of *Staphylococcus aureus* are required for animal infections, inhibitors that disrupt the activity of sortases may be therapeutically useful.

The conference was concluded with a description of the severe acute respiratory syndrome (SARS) epidemic in China. China's handling of the epidemic has been critiqued by the international community, but a balanced view of events has shown that many measures undertaken by officials, such as large-scale quarantines, mandatory fever-screening checkpoints, arrival and departure monitoring at airports, populationwide surveillance, community infection control, and designating SARS-only hospitals, worked effectively to contain this infectious disease.

The proceedings are available in PDF format at http://www.cvm.uiuc.edu/czr/

